# Raspberry ketone diet supplement reduces attraction of sterile male Queensland fruit fly to cuelure by altering expression of chemoreceptor genes

**DOI:** 10.1038/s41598-021-96778-7

**Published:** 2021-09-03

**Authors:** Mohammed Abul Monjur Khan, Nandan P. Deshpande, Lucas A. Shuttleworth, Terry Osborne, Damian Collins, Marc R. Wilkins, Geoff M. Gurr, Olivia L. Reynolds

**Affiliations:** 1 New South Wales Department of Primary Industries, Elizabeth Macarthur Agricultural Institute, Private Bag 4008, Narellan, NSW 2567 Australia; 2 Department of Entomology, Faculty of Agriculture, Bangladesh Agricultural University, Mymensingh, 2202 Bangladesh; 3 Systems Biology Initiative, School of Biotechnology and Biomolecular Sciences, University of New South Wales, Sydney, NSW 2052 Australia; 4 Ramaciotti Centre for Genomics, The University of New South Wales, Sydney, NSW 2052 Australia; 5 Graham Centre for Agricultural Innovation, Charles Sturt University, PO Box 883, Orange, NSW 2800 Australia; 6 Susentom, Heidelberg Heights, Melbourne, VIC 3081 Australia

**Keywords:** Ecology, Molecular biology, Zoology

## Abstract

Sterile male Queensland fruit fly, *Bactrocera tryoni* (Froggatt), fed as immature adults on the plant compound raspberry ketone (RK), show a reduced attraction to cuelure, a synthetic analogue of RK used as an attractant in Male Annihilation Technique. We hypothesized the reduced attraction of RK-fed adult males to cuelure may be a consequence of altered expression of chemoreception genes. A Y-tube olfactometer assay with RK-fed and RK-unfed sterile *B. tryoni* males tested the subsequent behavioural response to cuelure. Behavioral assays confirmed a significant decrease in attraction of RK-fed sterile males to cuelure. RK-fed, non-responders (to cue-lure) and RK-unfed, responders (to cue-lure) males were sampled and gene expression compared by de novo RNA-seq analysis. A total of 269 genes in fly heads were differentially expressed between replicated groups of RK-fed, cuelure non-responders and RK-unfed, cuelure responders. Among them, 218 genes including 4 chemoreceptor genes were up regulated and 51 genes were down regulated in RK-fed, cuelure non-responders. De novo assembly generated many genes with unknown functions and no significant BLAST hits to homologues in other species. The enriched and suppressed genes reported here, shed light on the transcriptional changes that affect the dynamics of insect responses to chemical stimuli.

## Introduction

Queensland fruit fly, *Bactrocera tryoni* (Froggatt) (Diptera: Tephritidae) is one of the most devastating insect pests of horticulture in Australia. It attacks almost all commercial fruits and many vegetables^1^, is a significant barrier to national and international horticultural market access^2^ and impacts the $15.1 billion Australian horticultural industry ^3^.

Sterile Insect Technique (SIT) and Male Annihilation Technique (MAT) are two of the most effective tools to manage Tephritidae pests^2^. In SIT programs, sterile males are released in the field to mate with fertile wild female populations, with a successful mating resulting in the production of non-viable offspring^4,5^. The number of sterile flies released in SIT programs can be modified based on the density of wild male individuals, which are most effective when wild population densities are low^6,7^. For this reason, MAT is used to reduce wild male numbers prior to SIT^2^. Cuelure-based MAT is a form of attract and kill for *B. tryoni*, which uses cuelure (a synthetic analogue of raspberry ketone) to attract male flies to feed, together with a toxicant (typically malathion) that when ingested results in mortality^8,9^.

The concurrent use of cuelure-based MAT and SIT to control *B tryoni* has been considered counterproductive because MAT devices capture the expensively produced sterile males. Recent studies, however have demonstrated that the concurrent use of MAT and SIT for *B. tryoni* may be feasible when the standard pre-release sterile male *B. tryoni* diet is supplemented with raspberry ketone (RK) to make them less strongly attracted to cuelure^10^. Studies with several other *Bactrocera* species show that pre-release feeding of males to plant-derived semiochemicals and synthetic lures dramatically reduces their subsequent response to MAT devices baited with these compounds^11–16^. However, the genetic basis for this effect has not been investigated, despite its relevance to the practical management of Tephritidae pests.

Insects perceive volatile compounds including the smell of lures through olfactory receptor neurons (ORNs) that are present in the olfactory sensilla and sensory hairs located in the antennae and maxillary palps^17^. Several proteins in insect olfactory sensilla such as odorant-binding proteins (OBPs), chemosensory proteins (CSPs) and odorant receptors (ORs) are involved in this chemoreception process^18,19^. Lipophilic odorant molecules in the environment reach the hydrophilic lymph of the insect through the micropores on the olfactory sensilla surface and then bind with OBPs or CSPs in the sensillary lymph to form a complex which is transported across the hydrophilic sensillum lymph that binds to the ORs on the dendritic membranes of ORNs^19^. When ORs are stimulated, the membrane permeability changes, resulting in the formation of an action potential that triggers cascade reactions, with the complex eventually entering the insect central nervous system. From this process, insects can sense exogenous odorant molecules and react accordingly to fulfill physiological responses^19^. Several OBPs and CSPs have been identified in various fruit fly species including the true fruit flies (Tephritidae) *Bactrocera dorsalis*^20^, Mediterranean fruit fly, *Ceratitis capitata* ^21,22^, *Anastrepha fraterculus* and *A. obliqua*^23^ and vinegar flies (Drosophilidae) *Drosophila melanogaster*^24,25^. Recent transcriptomic research has identified genes involved in the biosynthesis of OBPs and CSPs in several *Bactrocera* species including *B. dorsalis*^26–28^, *Bactrocera minax*^29^ and *B. tryoni*^30^.

It is now evident that host plant quality, phytochemicals and semiochemicals modulate expression of OBP and CSP genes in fruit flies, which influence the subsequent physiological behaviour including lure responsiveness, sexual calling and mating success^30–34^. In order to explore the genetic basis for these behavioural responses, we hypothesized that the decreased responsiveness of RK-fed sterile *B. tryoni* males to cuelure^10^ is associated with a variation in the expression of OBPs and CSPs that are responsible for olfaction^35^. The objective of this study was first to investigate the behavioural response of RK-fed and RK-unfed sterile male *B. tryoni* to cuelure using a Y-tube olfactometer, and then to investigate whether the genes of RK-fed non-responders and RK-unfed responders were associated with changes in the transcriptome.

## Results

### Y-tube olfactometer bioassay

**Figure 1:**
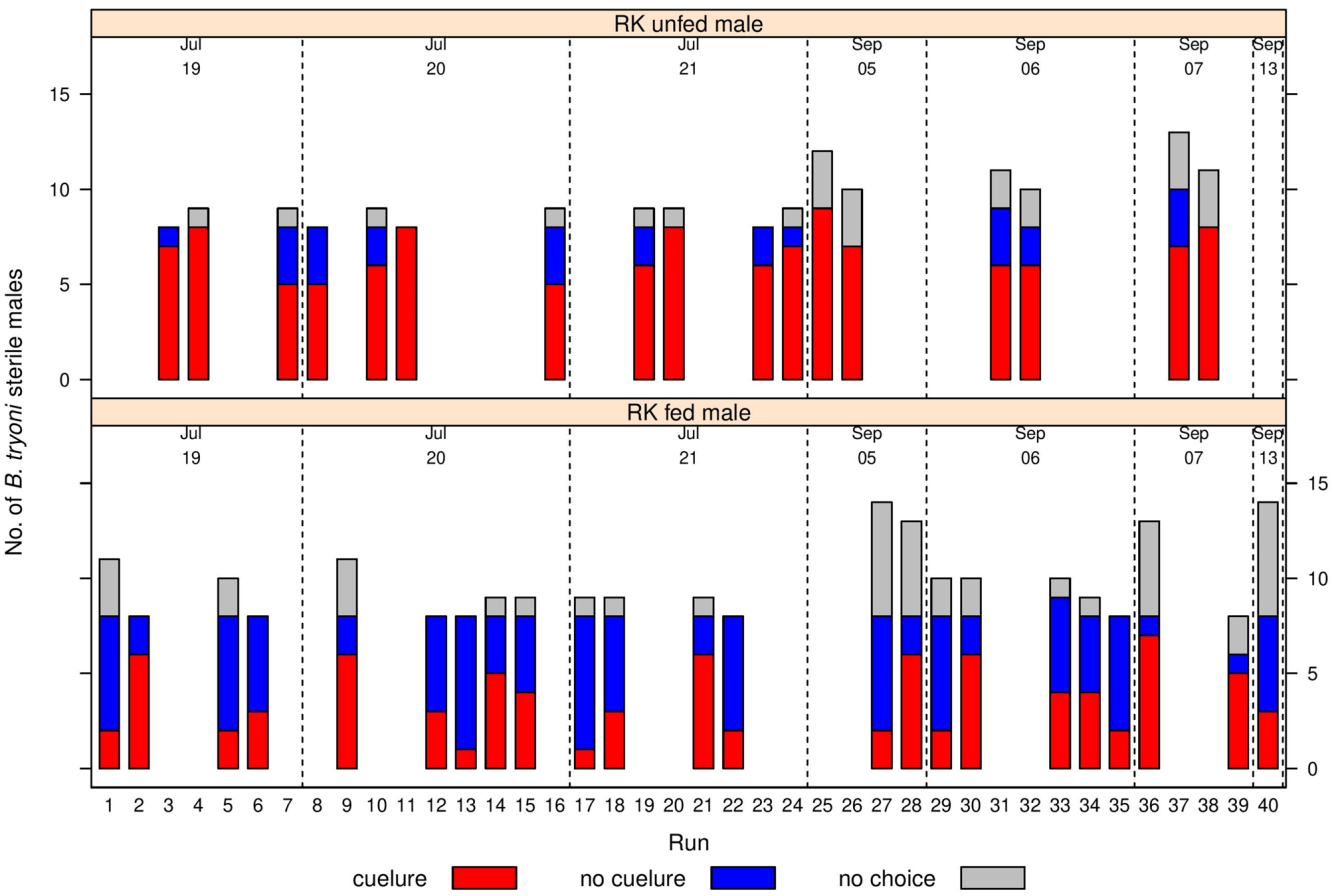
Y-tube olfactometer raw choice data for sterile male *B. tryoni* that were RK-unfed (top) or RK-fed (bottom) across 40 runs. Each bar shows the number of males selecting cuelure (red), no cuelure (blue) or no choice (grey). The trials were all run in 2018, with the date of each run shown above the bars, separated by vertical dotted lines.

**Figure 2:**
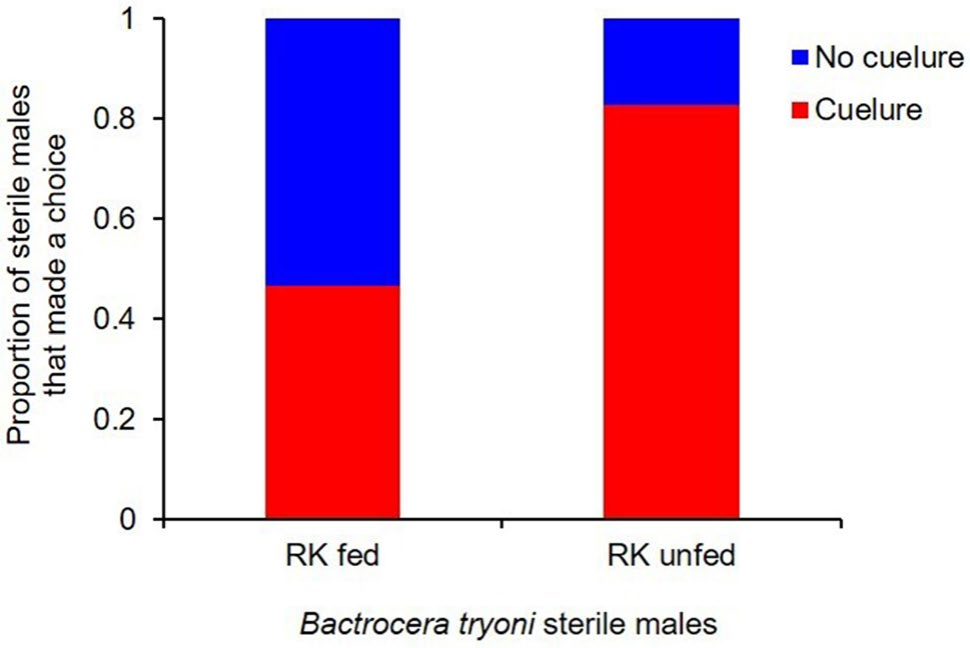
Total proportion of *B. tryoni* sterile males that made a choice in Y-tube olfactometer when exposed to cuelure. A significantly higher proportion of RK unfed sterile males chose the cuelure-baited arm compared with RK fed sterile males (p < 0.001).

**Figure 3:**
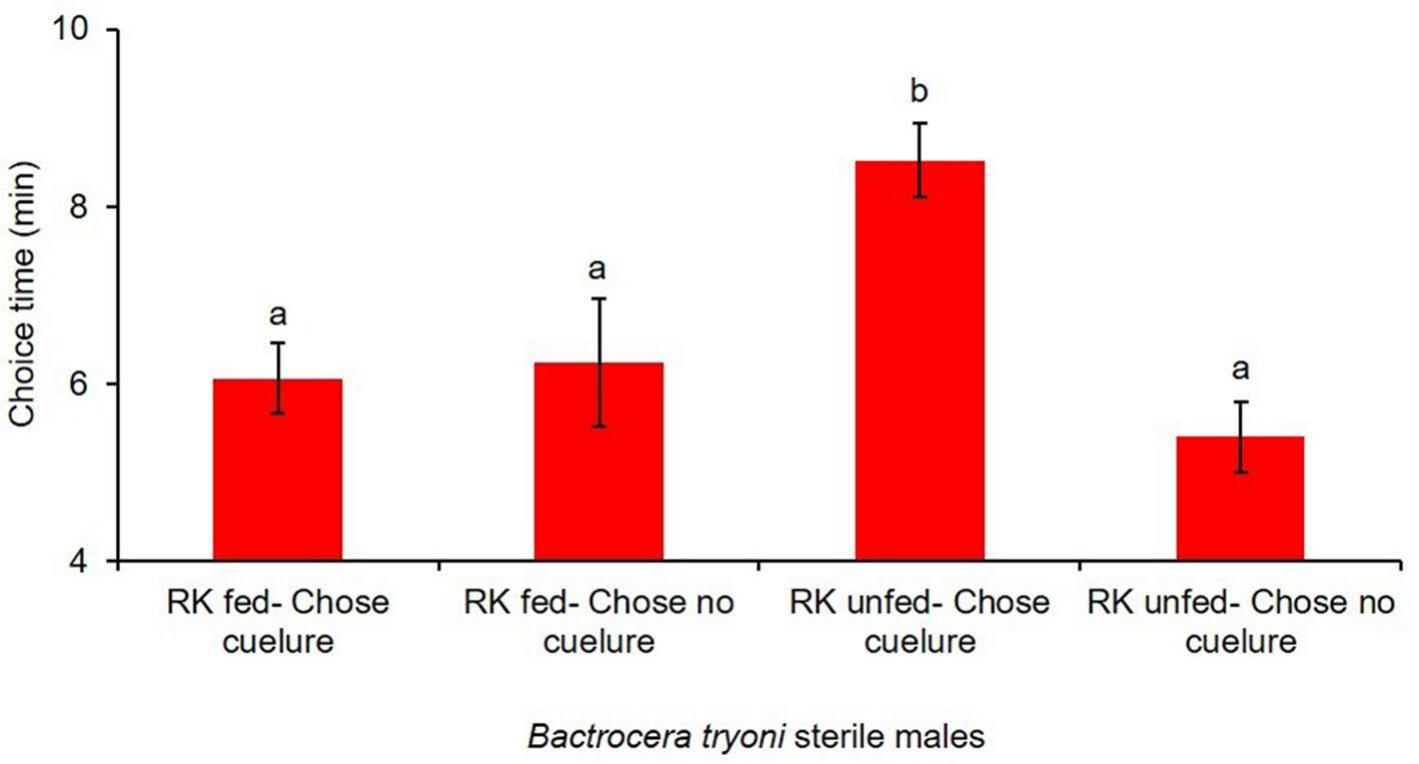
Time required by RK fed and RK unfed sterile male *B. tryoni* to make a choice inside the Y-tube olfactometer when exposed to cuelure. Lowercase letters on each column that differ are significantly different (p < 0.05).

There was a significant effect of feeding RK on the choice made by sterile male *B. tryoni* when exposed to cuelure in the Y- tube olfactometer (Fig. 1). RK fed males were significantly less attracted to cuelure (F_1,42.4_ = 25.2, p < 0.001) (Fig. 2) compared to RK unfed males. Among the male flies that made a choice, the proportion of RK unfed that chose the cuelure sourced arm was 0.83 ± 0.04, and the proportion of RK fed that responded to cue-lure was almost half that at 0.47 ± 0.05 (Fig. 2). The required time to make a choice by sterile males *B. tryoni* inside the olfactometer showed a large difference between treatments (F_1, 51_ = 4.50, P = 0.0389). RK unfed sterile males took a longer time to choose the cue lure sourced arm (8.52 ± 0.040 min) than the RK fed sterile males (6.06 ± 0.042 min) (Fig. 3).

### Transcriptomics of the RK-fed and RK-unfed male *B. tryoni*

RNA extracted from fly heads permitted four libraries to be generated for each of the RK-fed, cuelure non-responding and RK unfed cuelure responding groups. RNA-seq yielded 44 to 64 million, 2 × 75 nt paired-end reads which gave a total of 434,535,954 sequencing reads. The number of read-pairs generated per library is given in Supplementary Table 1.

The total number of raw contigs generated in the combined assembly using all read-pairs across all libraries was 52,651. The tool CD-HIT was used to reduce the redundancy to generate 30,137 clusters with 50,547,055 assembled bases. The average contig length was 1677 bp and the median contig length was found to be 1077 bp. The N50 parameter, which estimates the contiguity of the transcriptome assembly, was computed to be 2384 bp. The GC content of assembly was 37.9%. BUSCO analysis suggested that the transcriptome assembly was largely complete when compared against 1,658 insect gene orthologous groups, with 88% of the BUSCO groups found to be present and complete (Supplementary Table 2). The tool TransDecoder identified open reading frames for 29,554 transcripts of which 21,421 were complete (72%), 4,618 were partial on the 5' end, 1,991 were partial on the 3' end and 1,524 contained internal ORFs (partial on both ends).

### Odorant binding and chemosensory genes

With the primary focus of this experiment on odorant related responses, a detailed search was performed for odorant binding protein (OBP) and chemosensory genes from the genome and transcriptome datasets available for multiple fly species such as *D. melanogaster*, *Bactrocera dorsalis*, *Ceratitis capitata*, *Anastrepha obliqua* and *Anastrepha fraterculus*. The orthology detection tool Proteinortho was used to identify the OBP and chemosensory gene orthologs in the transcriptome assembly. A non-redundant set of 139 OBP/chemosensory-related gene orthologs were annotated.

### Differentially expressed genes (DEGs) between RK-fed, non-responding and RK unfed, responding flies

**Figure 4:**
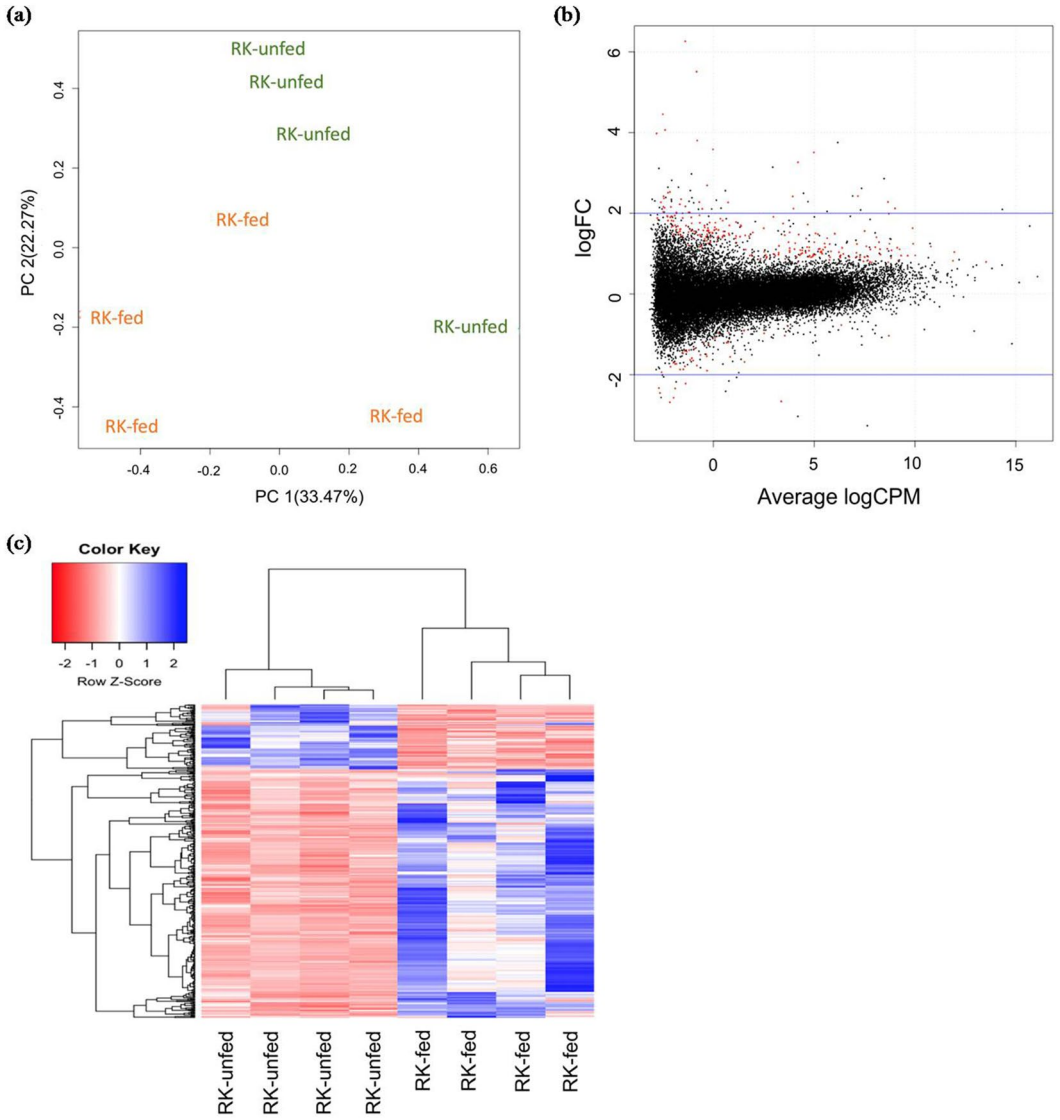
Differentially expressed genes (DEGs) in RK-fed, cuelure non-responding and RK-unfed, cuelure responding males. (**a**) Principal component analysis shows that colour-coded replicates show separation between conditions. (**b**) The expression profile of individual samples was explored with mean-difference (MD) plots. The differentially expressed genes are highlighted in red. The blue guidelines mark the log fold changes between ± 2. (**c**) Heatmap of all DEGs from all samples, with unsupervised hierarchical clustering on X and Y axes; drawn with coolmap function [https://rdrr.io/bioc/limma/man/coolmap.html] from bioconductor limma (3.48.1) package^59^. Clustering of samples was evident, as was clustering of genes that had correlated expression patterns.

Two hundred and sixty-nine genes were found to be differentially expressed between replicated groups of RK-fed, cuelure non-responding and RK-unfed, cuelure responding flies (Fig. 4). A total of 218 genes were significantly up regulated and 51 significantly down regulated in RK fed males (Supplementary Table 3). Of these, a very specific response was present in chemoreceptor genes, with only four showing up-regulation for the RK-fed, non-responding flies: putative gustatory receptor 39b, odorant-binding protein 99c isoform A, odorant binding protein 56a and CG4757 isoform A. The up-regulated genes also included multiple ribosomal protein isoforms, three Gram-negative bacteria binding proteins and three cuticular proteins. The gene pickpocket 17 (ppk17), isoform B, was highly down regulated (-5.9371FC) in RK-fed, non-responders. The other genes found to be down regulated in RK-fed non-responders include a protein kinase, cAMP-dependent, regulatory subunit type 2, isoform E, a shaking B, isoform D transcript and a glutaminase, isoform F transcript. Many genes with unknown functions and no significant BLAST hits to other species were also detected in the list of differentially expressed genes. Functional enrichment analysis of differentially expressed genes using Blast2GO did not reveal any specific categories.

### Discussion

The male annihilation technique (MAT) and sterile insect technique (SIT) are two important tools often used to control *B. tryoni* in Australia^9^. However, simultaneous use of MAT and SIT is counterproductive, because the MAT device baited with a male-specific lure would greatly reduce the number of costly sterile males that are released as part of an SIT program. A recent study demonstrated that the attraction of sterile male *B. tryoni* to cuelure baited MAT can be decreased when the sterile males are supplemented with RK prior to release^10^. The present study showed the reduced responsive behavior of RK fed sterile male *B. tryoni* to cuelure using a Y-tube olfactometer assay. Further, we showed, for the first time, a significant change in transcript levels associated with the chemo reception process in RK-fed cuelure non-responding *B. tryoni* sterile males through RNAseq analysis.

The Y-tube olfactometer assay in the current study confirmed a distinct reduced response to cuelure of RK fed sterile *B. tryoni* males under controlled conditions (devoid of other volatiles). The majority of the released RK fed males moved to the non-cuelure arm of the olfactometer, suggesting a distinct avoidance movement of the lure fed males from the cuelure. This finding supports earlier field-based trials, where a decreased proportion of RK-fed sterile male *B. tryoni* are attracted to cuelure-baited traps^10^. Indeed, in the field cage trial, RK-supplemented male *B. tryoni* exhibited a reduction in attraction to cuelure traps that lasted more than 20 days^36^.

To identify genes affected by RK in the cuelure response, we needed an appropriate reference transcriptome. Expression profiling analysis for non-model species such as *B. tryoni* require a *de-novo* assembly approach in order to capture the complete repertoire of transcripts expressed under specific experimental conditions. The draft genome assembly work by Gilchrist et al.generated a transcriptome of *B. tryoni*^37^. However, the draft transcriptome remains unpublished. The only publicly available *B. tryoni* transcriptome was generated using total mRNA obtained from the whole body of female flies^32^. In the absence of a more relevant transcriptome for our study, it was essential to generate a high quality de novo assembly. With a specific focus on the OBP and chemosensory genes, total mRNA was obtained from whole heads including antennae, of male *B. tryoni*. A transcriptome was generated with an N50 of 2,384 bp, with > 72% complete open reading frames and a BUSCO completeness percentage of 88%. These numbers highlight the utility of this transcriptome assembly for expression studies, as does the existence of 139 OBP/chemosensory-related orthologs identified in the transcriptome.

Insect OBPs and CSPs are responsible for the detection of host plant volatiles or sex pheromones in the environment^38,39^. Recent studies have demonstrated that adult dietary quality, exposure to volatile compounds, and copulation impact the expression profile of OBPs and CSPs in several fruit fly species. Oriental fruit fly, *B. dorsalis,* exposed to attractive protein baits and volatiles from brewer’s yeast showed significantly different transcript expression profiles, and showed high expression of several OBPs including OBP2, OBP5 and OBP1^33^. Female *B. tryoni* mated with zingerone-fed males had different OBP expression profiles at the post-mating stage, while OBP 56a and genes encoding cuticle proteins were down regulated^32^. In contrast, zingerone-fed males had up regulated transcripts including OBP, OBP 3, OBP22 and OBP56a, OBP 99c^30^. In the present study, a significant change in transcript levels was found in RK-fed *B. tryoni* males, compared to RK-unfed males when exposed to cuelure. A putative gustatory receptor 39b, in addition to OBP 99c, isoform A, OBP 56a and CG4757 were up-regulated in the RK-fed, cuelure non-responsive sterile males. Further, genes encoding cuticle proteins were up-regulated. It is possible that the up-regulation of these chemoreception-related genes might be associated with enhanced olfaction of cuelure that may have influenced the behaviour of RK-fed *B. tryoni* males, leading them to avoid the cuelure-associated arm of the olfactometer in order to evade repeated feeding on cuelure. Other *Bactrocera* species show a similar decreased repeated response to their respective synthetic lures, once they have fed on related plant-derived semiochemicals^13,40^.

In addition to the up-regulation of chemoreceptor genes, we identified changes in the expression of a member of the pickpocket (ppk) family of sodium channels, *ppk17* isoform B. This was highly down-regulated in RK-fed cuelure non-responders. The ppk family genes has an important role in taste perception^41^ and olfactory response in insects^35^. A *ppk* deficient mutant, such as *ppk11* mutant, affects olfactory response to benzaldehyde^42^. The *ppk25* is expressed and functions in neurons that detect female-specific pheromones, and males with impaired *ppk25* function court females at reduced rates^43^. In *Drosophila*, *ppk23* and *ppk29* are expressed in fruitless-positive neurons on the legs and are essential for courtship^44^. In our study, we showed that the *ppk17* isoform B, identified in *B. tryoni* may have a role in cuelure olfaction since this gene is down regulated in RK fed cuelure non-responsive males. Further functional genetic studies are warranted to confirm this and provide additional details on its function.

## Conclusion

This study generated a high quality transcriptome of male *B. tryoni* using whole heads including antennae, and identified the existence of OBP/chemosensory-related gene orthologs. The transcriptome will be useful not only in *B. tryoni* olfaction but could be used to identify new genes, transcription sites, and differentially expressed genes, as well as obtain functional gene information and transcription expression abundance. The data can be used for molecular marker development, gene expression analysis, and small RNA analysis. Differentially expressed genes, both up regulated and down regulated, identified in RK-fed and RK-unfed male flies can help understand factors possibly regulating cuelure selection behaviour of the lure-fed males and guide future functional studies on olfactory and chemosensory genes.

## Materials and methods

### Preparation of diet

An agar-based diet for adult *B. tryoni*^10^ was prepared with and without RK. The diet consisted of powdered agar (4.5 g), sugar (50 g), yeast hydrolysate enzymatic (60% protein, MP Biomedical, LLC, OH, USA) (20 g), and water (500 mL). The agar and sugar were first dissolved in water and boiled for 5–10 min, then allowed to cool to 40 °C, before the yeast was incorporated. A raspberry ketone solution (30%) was prepared by dissolving 30 g of RK powder (99%, Sigma Aldrich, USA) in 70 mL ethanol. A 1% RK supplement diet was selected, as previous studies showed increased survival, mating performance, and a decrease in the attraction of mature males to cuelure using this rate^10,45^. The 1% RK supplement diet was prepared by mixing 96.67 g of the agar diet with 3.33 mL of the RK solution with a hand held blender (Philips ProMix Hand blender, HR1686/98). The non RK diet was prepared by mixing 97.67 g of agar diet with 2.33 mL ethanol without RK. The prepared 100 g of diet was placed in two Petri dishes.

### Feeding regime for sterile male *B. tryoni*

Sterile *B. tryoni* pupae were sourced from the Fruit Fly Production Facility, Elizabeth Macarthur Agricultural Institute, Menangle, New South Wales (NSW), Australia. Pupae were placed on a Petri dish (60 mm × 15 mm) in two Bugdorm cages (30 × 30 × 30 cm; Megaview Science Co Ltd, Taiwan) at 26 ± 1ºC and 65 ± 10% relative humidity with a 14:10 h photoperiod^10^.One group of newly emerged adults (age 0–24 h) were provided with water and 100 g of agar diet containing 1% RK as the treatment and the other group received water and 100 g of agar diet without RK as the control, and permitted to feed for 48 h. Subsequently, female *B. tryoni* were separated and discarded and males were reared until 10 days old when flies were mature and responsive to cue-lure^46^. During this time all control and treated male *B. tryoni* were provided with yeast hydrolysate, sugar and water ad libitum*.*

### Y-tube olfactometer bioassay

Y-tube olfactometer bioassays were carried out as described in Najar-Rodriguez et al.^47^. The Y-tube olfactometer consisted of a Y-shaped glass tube (2.5 cm diameter, 23 cm arm length and 23 cm common arm length) connected to two tubular glass chambers (38 cm long and 6 cm in diameter) (OLFM-YT-2425F; Analytical Research Systems, Gainesville, Florida, USA), where the odor sources were placed (one in each arm). The odor sources consisted of (a) 500 μL of cuelure (Supelco, Bellefonte, PA, USA) pipetted onto cotton dental wicks loaded into an open-ended plastic cup (about 10 mm in diameter), and (b) clean air only (blank cotton dental wicks inside the plastic cup). A constant air flow rate of 300 ± 10 mL min^–1^ was maintained through each arm using an air compressor (ProjectAir, Model No. TA-COMP20, Spear & Jackson PTY Ltd, Victoria, Australia) and the air flow rate was monitored with a flow meter (Agilent flow meter ADM 1000; Agilent Technologies, Centerville, DE, USA). Prior to the bioassays, sterile male *B. tryoni* in groups of 100 from each diet treatment were acclimated in the experimental room for 30 min. A single male *B. tryoni* was released at the entrance of the common arm of the Y-tube and observed for 10 min. A choice was recorded when the fly entered one of the arms and crossed a score line drawn 3 cm from the intersection of the tube. A choice was not recorded if it remained in the common arm of the Y-tube by the end of the observation period^48^. The position of the arm of Y-tube was changed after testing 10 flies in order to avoid positional bias. After each 20 flies were tested, all parts of the olfactometer in contact with the flies was washed in a detergent solution, double rinsed with ethanol, and oven dried for 20 min at 150 °C. Y-tube olfactometer bioassays were carried out with a total of 40 runs across different days from 9.00 am to 5.00 pm, comprising 23 runs with RK fed- and 17 runs with RK unfed- sterile males, with the number of males tested in each run ranging between 8–14.

### Statistical analyses

A logistic mixed model was fitted to choice (binary response, with "no choice" ignored) with fixed effects of treatment (RK fed/RK-unfed) and random effects of runs (1–40). Time of day was also examined, but no significant effect was found on choice, therefore was excluded. For choice time, a logarithmic transformation was used, and a linear mixed model was fitted with fixed treatment effects, choice and their interaction, and random effects of run and run by choice. Reported means were back-transformed with approximate SE. All models were fitted in ASReml-R^49^.

### Transcriptomics

#### Sample preparation (sterile male *B. tryoni* heads)

The two types of sterile male flies of interest from the Y- tube olfactometer experiment were the RK-unfed, cuelure responders, and the RK-fed, cuelure non-responders, and hence these were the only specimens collected for RNA extraction. After individual flies made a choice in the Y-tube olfactometer, fly heads including antennae were excised and placed in 1.5 ml Eppendorf tubes and immediately stored on dry ice (~ -80 °C). Fifteen heads were pooled together in a single tube, representing one biological sample for each fly type. A total of four biological samples were prepared for each tube of RK-unfed, cuelure responders and RK-fed, cuelure non-responder male flies. The fly heads samples were transferred to − 80 °C for storage until analysis.

#### RNA extraction

Total RNA was extracted from each tissue sample and purified using a RNeasy kit (Qiagen, Hilden, Germany) following the manufacturer’s instructions. Total RNA was quantified using a NanoDrop spectrophotometer (Thermo Scientific, Wilmington, DE,USA) and quality checked using electrophoresis through a 1.1% agarose gel. Messenger RNA (mRNA) was isolated from total RNA using a PolyAttract mRNA Isolation System III (Promega, Madison, WI, USA). The mRNAs were sheared into approximately 800 nucleotide lengths via RNA fragmentation solution, cleaned and condensed using a RNeasy MinElute Cleanup Kit (Qiagen, Valencia, CA, USA).

#### Transcriptome sequencing, assembly and functional annotation

Libraries were prepared using the TruSeq Stranded mRNA-seq kit (Illumina). One µg of RNA was used as input for the poly-A pulldown followed by cDNA synthesis, A-tailing and adapter ligation. The libraries were enriched using 12 PCR cycles and sequenced on the NextSeq 500 platform using the High Output v2 kit and 2 × 75 bp reads. The raw reads were quality trimmed using Trimmomatic^50^ with default parameters, and only high-quality reads were retained for assembly. The transcriptomes were assembled using the Trinity de novo assembler v2.3.2^51^. The tool CD-Hit^52^ was used to remove redundant and chimeric sequences. Sequences with greater than 95% similarity were clustered together. The tool BUSCO (Benchmarking Universal Single-Copy Orthologs)^53^ was used to determine the percentage of full-length sequences in the assembled transcriptome, by comparison with 1658 highly conserved insect proteins. The tool TransDecoder (https://github.com/TransDecoder/TransDecoder/wiki) was used to identify candidate coding regions within transcript sequences. Functional annotation of the transcriptome was done using the tool Blast2GO^54^ by using BLASTx searches against the NCBI nr database and specifically against the *Drosophila melanogaster* protein sequences.

#### Read mapping, quantification and differential expression analysis

The tool bwa^55^ was used to map individual samples to the assembled reference transcriptome. Salmon^56^, an ultra-fast tool for transcript quantification from RNA-seq data was then employed for read quantification. The R package RUVSeq^57^ was used to conduct a differential expression (DE) analysis that controls for and removes unwanted variation e.g., batch, library preparation, and other nuisance effects, using between-sample normalization methods. The differential expression identification step was conducted using the negative binomial GLM approach implemented in the R- package edgeR^57^. A false discovery rate (FDR) of ≤ 0.05 was used to determine statistically significant differential expression in the comparison of RK-fed, cuelure non-responsive versus RK-unfed, cuelure responsive male samples. Gene enrichment analysis of the DE genes was done using the FatiGO^58^ package inside the Blast2GO environment. The FatiGO package uses the Fisher's Exact Test and corrects for multiple testing. The list of enriched Gene Ontologies was reduced to more specific terms.

## Supplementary Information

Supplementary Information.

## Data Availability

The RNA‐Seq raw data have been deposited in the European Nucleotide Archive (ENA) with accession number ERS3223683. The transcriptome has been submitted to the ENA with accession number PRJEB31685, and publicly available.
