# Supplementary material for: Raspberry ketone diet supplement reduces attraction of sterile male Queensland fruit fly to cuelure by altering expression of chemoreceptor genes
Source: Sci Rep. 2021 Sep 3;11:17632. doi: 10.1038/s41598-021-96778-7 (PMC8417256; doi:10.1038/s41598-021-96778-7)
Supplement: Supplementary file 1 — Supplementary Information. [file 41598_2021_96778_MOESM1_ESM.docx]

**Supplementary Table 1.** An average of 54 million reads was generated per sample, across 8 samples with 4 RK-fed and 4 RK-unfed male *B. tryoni* replicates.

| **Sample No.** | **Sample type** | **Read-pair (*2)** |
| --- | --- | --- |
| 1. | RK unfed | 59,206,227 |
| 2. | RK unfed | 64,360,787 |
| 3. | RK unfed | 53,161,380 |
| 4. | RK unfed | 59,050,089 |
| 5. | RK fed | 52,622,293 |
| 6. | RK fed | 44,223,865 |
| 7. | RK fed | 50,771,723 |
| 8. | RK fed | 51,139,590 |
|  | **Average** | **54,316,994** |

**Supplementary Table 2.** BUSCO statistics showed a high level of completeness of the transcriptome assembly with only 7.1% BUSCO groups missing.

| Summarized benchmarks in BUSCO notation against insect groups:  C:88.3% [D:11%], F:4.6%, M:7.1%, n:1658  1464 Complete BUSCOs  1282 Complete and single-copy BUSCOs  182 Complete and duplicated BUSCOs  76 Fragmented BUSCOs  118 Missing BUSCOs  1658 Total BUSCO groups searched |
| --- |

**Supplementary Table 3.** List of differentially expressed transcripts identified using the tool EdgeR with a FDR cut-off of FDR ≤ 0.05. The RK-unfed responsive datasets were used as controls.

| **Transcript ID** | **logFC** | **FC** | **P Value** | **FDR** | **Description**  (where applicable) |
| --- | --- | --- | --- | --- | --- |
| TRINITY_DN13398_c0_g1_i1\|len=1377 | 0.9980 | 1.9972 | 0.0001 | 0.0222 | putative gustatory receptor 39b |
| TRINITY_DN13206_c0_g1_i1\|len=649 | 1.1832 | 2.2708 | 0.0000 | 0.0015 | odorant binding protein 99c, isoform A |
| TRINITY_DN16428_c0_g1_i2\|len=718 | 1.1071 | 2.1541 | 0.0001 | 0.0183 | odorant binding protein 56a |
| TRINITY_DN17751_c0_g1_i1\|len=2909 | 0.8906 | 1.8540 | 0.0004 | 0.0470 | CG4757, isoform A |
| TRINITY_DN16512_c0_g1_i1\|len=991 | 1.1693 | 2.2490 | 0.0002 | 0.0275 | cuticular protein 72Eb |
| TRINITY_DN20137_c0_g1_i6\|len=655 | 2.2764 | 4.8447 | 0.0001 | 0.0231 | cuticular protein 5C |
| TRINITY_DN4449_c0_g1_i1\|len=563 | 1.1024 | 2.1471 | 0.0001 | 0.0176 | cuticular protein 47Eg |
| TRINITY_DN25113_c0_g1_i1\|len=722 | 1.5851 | 3.0004 | 0.0002 | 0.0290 | ribosomal protein S9, isoform E |
| TRINITY_DN24253_c0_g1_i1\|len=764 | 1.5002 | 2.8289 | 0.0001 | 0.0157 | ribosomal protein S5a, isoform B |
| TRINITY_DN859_c0_g1_i1\|len=692 | 1.8169 | 3.5233 | 0.0000 | 0.0027 | ribosomal protein S27A |
| TRINITY_DN9007_c0_g1_i3\|len=1446 | 2.0229 | 4.0640 | 0.0000 | 0.0076 | ribosomal protein S24 |
| TRINITY_DN3357_c0_g1_i1\|len=505 | 0.8361 | 1.7853 | 0.0005 | 0.0473 | ribosomal protein S21, isoform F |
| TRINITY_DN27639_c0_g1_i1\|len=698 | 1.4797 | 2.7890 | 0.0002 | 0.0255 | ribosomal protein S20 |
| TRINITY_DN4471_c0_g1_i1\|len=865 | 1.4570 | 2.7454 | 0.0003 | 0.0390 | ribosomal protein S16, isoform B |
| TRINITY_DN6502_c0_g1_i1\|len=597 | 1.5656 | 2.9601 | 0.0000 | 0.0102 | ribosomal protein S15Aa, isoform F |
| TRINITY_DN27795_c0_g1_i1\|len=1105 | 1.5512 | 2.9305 | 0.0001 | 0.0210 | ribosomal protein L5, isoform A |
| TRINITY_DN1045_c0_g1_i1\|len=547 | 1.4239 | 2.6831 | 0.0002 | 0.0310 | ribosomal protein L34b, isoform C |
| TRINITY_DN24542_c0_g1_i1\|len=627 | 1.7020 | 3.2534 | 0.0001 | 0.0186 | ribosomal protein L32, isoform D |
| TRINITY_DN26240_c0_g1_i1\|len=539 | 2.1102 | 4.3176 | 0.0000 | 0.0015 | ribosomal protein L31, isoform B |
| TRINITY_DN673_c0_g1_i1\|len=552 | 1.7701 | 3.4108 | 0.0000 | 0.0053 | ribosomal protein L30, isoform E |
| TRINITY_DN26702_c0_g1_i1\|len=542 | 1.5845 | 2.9990 | 0.0000 | 0.0127 | ribosomal protein L27A, isoform C |
| TRINITY_DN2785_c0_g1_i1\|len=513 | 1.4673 | 2.7651 | 0.0001 | 0.0209 | ribosomal protein L22 |
| TRINITY_DN27477_c0_g1_i1\|len=692 | 1.6024 | 3.0365 | 0.0001 | 0.0230 | ribosomal protein L21, isoform B |
| TRINITY_DN24648_c0_g1_i1\|len=806 | 1.5234 | 2.8746 | 0.0001 | 0.0222 | ribosomal protein L18, isoform B |
| TRINITY_DN2560_c0_g1_i1\|len=622 | 1.5684 | 2.9657 | 0.0000 | 0.0127 | ribosomal protein L12, isoform A |
| TRINITY_DN4289_c0_g1_i1\|len=794 | 1.4724 | 2.7748 | 0.0002 | 0.0294 | Gram-negative bacteria binding protein 3 |
| TRINITY_DN20272_c0_g2_i1\|len=1550 | 1.0244 | 2.0341 | 0.0001 | 0.0230 | Gram-negative bacteria binding protein 1 |
| TRINITY_DN20272_c0_g1_i1\|len=1808 | 0.9980 | 1.9973 | 0.0003 | 0.0329 | Gram-negative bacteria binding protein 1 |
| TRINITY_DN18724_c0_g1_i1\|len=1482 | 1.0481 | 2.0678 | 0.0002 | 0.0309 | vermilion |
| TRINITY_DN24936_c0_g1_i1\|len=1049 | 1.9460 | 3.8529 | 0.0001 | 0.0204 | vacuolar protein sorting 26 |
| TRINITY_DN7041_c0_g1_i2\|len=1020 | 1.6611 | 3.1627 | 0.0004 | 0.0425 | UDP-galactose 4'-epimerase, isoform B |
| TRINITY_DN633_c0_g1_i1\|len=1222 | 1.5236 | 2.8751 | 0.0002 | 0.0317 | Tryptophanyl-tRNAsynthetase |
| TRINITY_DN12861_c0_g1_i1\|len=1245 | 1.1490 | 2.2177 | 0.0000 | 0.0072 | trypsin 29F, isoform D |
| TRINITY_DN22098_c0_g1_i6\|len=4598 | 1.4327 | 2.6995 | 0.0003 | 0.0405 | trynity, isoform A |
| TRINITY_DN13756_c0_g1_i1\|len=726 | 1.1365 | 2.1984 | 0.0004 | 0.0415 | triforce, isoform B |
| TRINITY_DN3063_c0_g1_i1\|len=528 | 2.2239 | 4.6714 | 0.0000 | 0.0015 | thioredoxin T, isoform B |
| TRINITY_DN19679_c0_g1_i1\|len=4079 | 0.7834 | 1.7212 | 0.0004 | 0.0415 | Thioester-containing protein 2, isoform F |
| TRINITY_DN4451_c0_g1_i1\|len=2444 | 4.0813 | 16.9271 | 0.0001 | 0.0182 | splicing factor 2, isoform B |
| TRINITY_DN772_c0_g1_i1\|len=724 | 0.9208 | 1.8931 | 0.0001 | 0.0234 | SIFamide |
| TRINITY_DN18737_c0_g1_i1\|len=3277 | 1.0347 | 2.0486 | 0.0002 | 0.0282 | Serine-peptidase 212 |
| TRINITY_DN17553_c0_g1_i2\|len=942 | 1.5590 | 2.9465 | 0.0000 | 0.0036 | serine protease 6 |
| TRINITY_DN17553_c0_g1_i1\|len=1498 | 1.2859 | 2.4383 | 0.0000 | 0.0111 | serine protease 6 |
| TRINITY_DN18397_c0_g1_i1\|len=1991 | 1.0605 | 2.0856 | 0.0001 | 0.0144 | scavenger receptor acting in neural tissue and majority of rhodopsin is absent, isoform C |
| TRINITY_DN3955_c0_g1_i1\|len=846 | 2.4336 | 5.4023 | 0.0001 | 0.0202 | RNA polymerase II 18kD subunit |
| TRINITY_DN24695_c0_g1_i1\|len=1178 | 1.4518 | 2.7356 | 0.0001 | 0.0157 | receptor of activated protein kinase C 1, isoform C |
| TRINITY_DN17345_c0_g1_i1\|len=1762 | 1.1881 | 2.2785 | 0.0002 | 0.0255 | prolyl-4-hydroxylase-alpha MP |
| TRINITY_DN21630_c0_g1_i1\|len=2059 | 1.1571 | 2.2301 | 0.0000 | 0.0111 | phosphoribosylamidotransferase 2, isoform B |
| TRINITY_DN25560_c0_g1_i1\|len=856 | 1.6334 | 3.1024 | 0.0000 | 0.0033 | phosphoglyceromutase, isoform C |
| TRINITY_DN16325_c0_g1_i1\|len=800 | 0.9938 | 1.9914 | 0.0001 | 0.0176 | PGRP-SB2, isoform A |
| TRINITY_DN7066_c0_g1_i1\|len=1198 | 1.3742 | 2.5922 | 0.0001 | 0.0165 | nimrod C3 |
| TRINITY_DN17095_c0_g1_i1\|len=1564 | 0.9906 | 1.9870 | 0.0004 | 0.0415 | nimrod B4 |
| TRINITY_DN19704_c0_g1_i1\|len=2382 | 0.9216 | 1.8942 | 0.0001 | 0.0183 | neprilysin 1, isoform C |
| TRINITY_DN3803_c0_g1_i2\|len=679 | 1.8980 | 3.7269 | 0.0000 | 0.0072 | mitochondrial pyruvate carrier, isoform C |
| TRINITY_DN2351_c0_g1_i1\|len=788 | 2.0229 | 4.0640 | 0.0000 | 0.0015 | magonashi |
| TRINITY_DN11140_c0_g1_i2\|len=600 | 2.4315 | 5.3947 | 0.0001 | 0.0157 | lysozyme E |
| TRINITY_DN6757_c0_g1_i2\|len=534 | 2.4152 | 5.3340 | 0.0001 | 0.0190 | lysozyme D |
| TRINITY_DN23477_c0_g1_i1\|len=1131 | 0.9628 | 1.9492 | 0.0000 | 0.0035 | lectin-24A |
| TRINITY_DN11935_c0_g1_i1\|len=1171 | 2.1190 | 4.3440 | 0.0000 | 0.0080 | lambdaTry |
| TRINITY_DN22358_c0_g1_i1\|len=2838 | 0.8688 | 1.8262 | 0.0005 | 0.0484 | ladybird late, isoform A |
| TRINITY_DN24360_c0_g1_i1\|len=678 | 1.6970 | 3.2423 | 0.0000 | 0.0116 | histone H2A |
| TRINITY_DN21111_c0_g1_i1\|len=1571 | 0.9526 | 1.9353 | 0.0000 | 0.0127 | henna, isoform C |
| TRINITY_DN12550_c0_g1_i1\|len=809 | 0.9098 | 1.8788 | 0.0001 | 0.0232 | Growth-blocking peptide 3, isoform B |
| TRINITY_DN19225_c0_g1_i1\|len=2278 | 1.0326 | 2.0457 | 0.0001 | 0.0231 | glutactin, isoform D |
| TRINITY_DN27327_c0_g1_i1\|len=1224 | 1.7529 | 3.3705 | 0.0000 | 0.0072 | fructose-1,6-bisphosphatase, isoform C |
| TRINITY_DN21836_c1_g1_i1\|len=2321 | 0.9055 | 1.8732 | 0.0003 | 0.0360 | framauro, isoform G |
| TRINITY_DN8461_c0_g1_i1\|len=1896 | 1.1064 | 2.1531 | 0.0002 | 0.0276 | esterase Q |
| TRINITY_DN1088_c0_g1_i1\|len=695 | 1.5940 | 3.0188 | 0.0002 | 0.0259 | eIF-5A, isoform A |
| TRINITY_DN603_c0_g1_i1\|len=705 | 1.7047 | 3.2595 | 0.0002 | 0.0280 | effete, isoform A |
| TRINITY_DN3881_c0_g1_i1\|len=656 | 0.7923 | 1.7319 | 0.0002 | 0.0309 | drosulfakinin |
| TRINITY_DN20117_c0_g1_i1\|len=1851 | 4.3907 | 20.9766 | 0.0000 | 0.0015 | doublesex-Mab related 99B |
| TRINITY_DN1070_c0_g1_i1\|len=666 | 1.3855 | 2.6126 | 0.0004 | 0.0415 | discs overgrown, isoform B |
| TRINITY_DN7489_c0_g1_i1\|len=1244 | 2.2720 | 4.8298 | 0.0001 | 0.0222 | dicarboxylate carrier 1, isoform C |
| TRINITY_DN9310_c0_g1_i1\|len=2712 | 1.2527 | 2.3829 | 0.0003 | 0.0341 | cytochrome P450-18a1, isoform B |
| TRINITY_DN7854_c0_g1_i1\|len=1785 | 1.0198 | 2.0276 | 0.0001 | 0.0231 | cytochrome b5-related, isoform B |
| TRINITY_DN11992_c0_g1_i1\|len=1970 | 0.9194 | 1.8913 | 0.0004 | 0.0460 | Cyp28d1 |
| TRINITY_DN3397_c0_g1_i2\|len=686 | 0.9142 | 1.8845 | 0.0001 | 0.0156 | cyclope, isoform B |
| TRINITY_DN16599_c0_g1_i1\|len=1876 | 0.9193 | 1.8912 | 0.0001 | 0.0147 | commonDpr-interacting protein |
| TRINITY_DN22035_c0_g1_i2\|len=2552 | 2.3443 | 5.0780 | 0.0002 | 0.0266 | commissureless, isoform B |
| TRINITY_DN12570_c0_g1_i1\|len=1979 | 1.0393 | 2.0552 | 0.0005 | 0.0498 | cinnabar |
| TRINITY_DN12849_c0_g1_i1\|len=1992 | 0.9134 | 1.8835 | 0.0001 | 0.0222 | CG8952 |
| TRINITY_DN19303_c0_g1_i1\|len=3140 | 1.4837 | 2.7967 | 0.0000 | 0.0035 | CG8774, isoform A |
| TRINITY_DN14242_c0_g1_i1\|len=1757 | 1.4367 | 2.7070 | 0.0000 | 0.0074 | CG7460, isoform C |
| TRINITY_DN6346_c0_g1_i1\|len=2142 | 0.8427 | 1.7934 | 0.0004 | 0.0458 | CG5991, isoform A |
| TRINITY_DN22065_c0_g1_i1\|len=3346 | 1.1922 | 2.2850 | 0.0001 | 0.0176 | CG5849, isoform A |
| TRINITY_DN19968_c1_g1_i1\|len=657 | 1.0992 | 2.1424 | 0.0004 | 0.0460 | CG5390, isoform B |
| TRINITY_DN18740_c1_g1_i8\|len=3011 | 1.8608 | 3.6321 | 0.0001 | 0.0222 | CG5065, isoform A |
| TRINITY_DN22663_c0_g1_i1\|len=2401 | 0.9712 | 1.9604 | 0.0001 | 0.0233 | CG4725 |
| TRINITY_DN19058_c0_g1_i5\|len=1072 | 2.1238 | 4.3584 | 0.0000 | 0.0015 | CG4653 |
| TRINITY_DN19058_c0_g1_i3\|len=904 | 2.2820 | 4.8636 | 0.0000 | 0.0015 | CG4053 |
| TRINITY_DN2941_c0_g1_i2\|len=1944 | 1.6040 | 3.0399 | 0.0004 | 0.0459 | CG3792 |
| TRINITY_DN20776_c0_g1_i1\|len=2439 | 1.2855 | 2.4376 | 0.0000 | 0.0019 | CG3775, isoform B |
| TRINITY_DN21271_c0_g1_i1\|len=2254 | 0.9120 | 1.8817 | 0.0004 | 0.0415 | CG3588, isoform F |
| TRINITY_DN19998_c0_g1_i3\|len=1685 | 0.9724 | 1.9621 | 0.0003 | 0.0324 | CG1299, isoform A |
| TRINITY_DN21937_c0_g1_i1\|len=1775 | 1.1111 | 2.1600 | 0.0000 | 0.0077 | CG1092, isoform D |
| TRINITY_DN15587_c1_g1_i1\|len=916 | 1.1810 | 2.2674 | 0.0000 | 0.0069 | cellular repressor of E1A-stimulated genes, isoform D |
| TRINITY_DN19798_c0_g1_i1\|len=2759 | 0.9614 | 1.9472 | 0.0003 | 0.0377 | brown, isoform D |
| TRINITY_DN18124_c0_g1_i2\|len=1239 | 3.2650 | 9.6133 | 0.0000 | 0.0123 | beta-site APP-cleaving enzyme, isoform A |
| TRINITY_DN13883_c0_g1_i1\|len=2519 | 1.0126 | 2.0176 | 0.0001 | 0.0149 | arginase |
| TRINITY_DN19535_c0_g1_i1\|len=2162 | 0.9589 | 1.9438 | 0.0001 | 0.0222 | Ance-4, isoform A |
| TRINITY_DN1902_c0_g1_i1\|len=1712 | 1.5214 | 2.8708 | 0.0004 | 0.0407 | alpha-Tubulin at 84B |
| TRINITY_DN19623_c0_g1_i2\|len=1990 | 1.1187 | 2.1716 | 0.0002 | 0.0309 | aldehyde dehydrogenase, isoform B |
| TRINITY_DN13829_c0_g1_i1\|len=1770 | 1.0739 | 2.1050 | 0.0001 | 0.0146 | adenosinedeaminase-related growth factor A, isoform A |
| TRINITY_DN4101_c0_g1_i2\|len=1267 | 1.3807 | 2.6040 | 0.0002 | 0.0253 | 14-3-3epsilon, isoform D |
| TRINITY_DN5593_c0_g1_i1\|len=1205 | 1.6543 | 3.1477 | 0.0000 | 0.0036 | uncharacterized protein Dmel_CG45050, isoform C |
| TRINITY_DN11343_c0_g1_i1\|len=761 | 0.9500 | 1.9319 | 0.0002 | 0.0315 | uncharacterized protein Dmel_CG34454 |
| TRINITY_DN21653_c0_g1_i2\|len=2649 | 0.9511 | 1.9333 | 0.0001 | 0.0225 | uncharacterized protein Dmel_CG34402, isoform C |
| TRINITY_DN9766_c0_g1_i1\|len=942 | 1.1560 | 2.2284 | 0.0001 | 0.0235 | uncharacterized protein Dmel_CG34177 |
| TRINITY_DN11144_c0_g1_i1\|len=1487 | 0.9245 | 1.8980 | 0.0001 | 0.0222 | uncharacterized protein Dmel_CG33493, isoform A |
| TRINITY_DN16682_c0_g1_i1\|len=1263 | 1.0363 | 2.0509 | 0.0000 | 0.0127 | uncharacterized protein Dmel_CG30280, isoform B |
| TRINITY_DN22002_c0_g1_i2\|len=2174 | 1.0586 | 2.0829 | 0.0003 | 0.0341 | uncharacterized protein Dmel_CG18249, isoform B |
| TRINITY_DN7601_c0_g1_i1\|len=1410 | 1.0752 | 2.1071 | 0.0000 | 0.0109 | uncharacterized protein Dmel_CG18003 |
| TRINITY_DN8318_c0_g1_i3\|len=850 | 1.0989 | 2.1419 | 0.0000 | 0.0072 | uncharacterized protein Dmel_CG16799, isoform B |
| TRINITY_DN23053_c0_g1_i1\|len=3422 | 1.0221 | 2.0309 | 0.0001 | 0.0231 | uncharacterized protein Dmel_CG16798 |
| TRINITY_DN6037_c0_g1_i3\|len=1072 | 3.1406 | 8.8190 | 0.0000 | 0.0072 | uncharacterized protein Dmel_CG14903 |
| TRINITY_DN23181_c0_g1_i1\|len=3198 | 1.2391 | 2.3605 | 0.0000 | 0.0089 | uncharacterized protein Dmel_CG14880, isoform A |
| TRINITY_DN996_c0_g1_i1\|len=1971 | 1.6305 | 3.0962 | 0.0000 | 0.0011 | uncharacterized protein Dmel_CG14606, isoform C |
| TRINITY_DN12367_c0_g1_i1\|len=716 | 1.4577 | 2.7468 | 0.0003 | 0.0368 | uncharacterized protein Dmel_CG13043 |
| TRINITY_DN16317_c0_g1_i1\|len=1238 | 1.1611 | 2.2362 | 0.0000 | 0.0030 | uncharacterized protein Dmel_CG11843, isoform B |
| TRINITY_DN18002_c0_g1_i1\|len=1742 | 1.3042 | 2.4696 | 0.0003 | 0.0377 | uncharacterized protein Dmel_CG11382, isoform C |
| TRINITY_DN15194_c0_g1_i3\|len=1245 | 8.7187 | 421.3107 | 0.0000 | 0.0005 | - |
| TRINITY_DN21927_c2_g2_i7\|len=1528 | 5.5241 | 46.0163 | 0.0000 | 0.0016 | - |
| TRINITY_DN7964_c0_g1_i2\|len=1073 | 4.4817 | 22.3419 | 0.0000 | 0.0033 | - |
| TRINITY_DN24948_c0_g1_i1\|len=506 | 3.9926 | 15.9179 | 0.0000 | 0.0014 | - |
| TRINITY_DN20953_c1_g1_i2\|len=1525 | 3.7884 | 13.8173 | 0.0000 | 0.0002 | - |
| TRINITY_DN16683_c0_g1_i2\|len=1818 | 3.5771 | 11.9349 | 0.0001 | 0.0215 | - |
| TRINITY_DN6308_c0_g1_i1\|len=627 | 3.5066 | 11.3653 | 0.0000 | 0.0115 | - |
| TRINITY_DN27023_c0_g1_i1\|len=627 | 2.8415 | 7.1674 | 0.0001 | 0.0232 | - |
| TRINITY_DN15449_c0_g1_i1\|len=932 | 2.6880 | 6.4440 | 0.0002 | 0.0309 | - |
| TRINITY_DN10453_c0_g1_i2\|len=1237 | 2.6518 | 6.2845 | 0.0002 | 0.0255 | - |
| TRINITY_DN1589_c0_g1_i1\|len=616 | 2.5258 | 5.7591 | 0.0000 | 0.0015 | - |
| TRINITY_DN967_c0_g1_i1\|len=728 | 2.5082 | 5.6892 | 0.0000 | 0.0013 | - |
| TRINITY_DN25444_c0_g1_i1\|len=670 | 2.3782 | 5.1990 | 0.0001 | 0.0222 | - |
| TRINITY_DN26646_c0_g1_i1\|len=540 | 2.2775 | 4.8485 | 0.0000 | 0.0009 | - |
| TRINITY_DN3566_c0_g1_i1\|len=723 | 2.1708 | 4.5027 | 0.0000 | 0.0033 | - |
| TRINITY_DN6572_c0_g1_i1\|len=539 | 2.1577 | 4.4621 | 0.0000 | 0.0079 | - |
| TRINITY_DN27947_c0_g1_i1\|len=880 | 2.1572 | 4.4604 | 0.0000 | 0.0053 | - |
| TRINITY_DN26562_c0_g1_i1\|len=807 | 2.1345 | 4.3909 | 0.0000 | 0.0115 | - |
| TRINITY_DN26501_c0_g1_i1\|len=708 | 2.1331 | 4.3865 | 0.0001 | 0.0187 | - |
| TRINITY_DN9063_c0_g1_i1\|len=739 | 2.0442 | 4.1244 | 0.0004 | 0.0469 | - |
| TRINITY_DN26135_c0_g1_i1\|len=594 | 2.0202 | 4.0565 | 0.0003 | 0.0405 | - |
| TRINITY_DN16674_c0_g1_i3\|len=723 | 2.0024 | 4.0066 | 0.0001 | 0.0176 | - |
| TRINITY_DN27349_c0_g1_i1\|len=829 | 1.9988 | 3.9966 | 0.0000 | 0.0083 | - |
| TRINITY_DN790_c0_g1_i1\|len=680 | 1.9529 | 3.8715 | 0.0002 | 0.0260 | - |
| TRINITY_DN28054_c0_g1_i1\|len=993 | 1.9259 | 3.7998 | 0.0003 | 0.0405 | - |
| TRINITY_DN26844_c0_g1_i2\|len=559 | 1.9177 | 3.7781 | 0.0003 | 0.0345 | - |
| TRINITY_DN25002_c0_g1_i1\|len=753 | 1.9142 | 3.7691 | 0.0000 | 0.0069 | - |
| TRINITY_DN11393_c0_g1_i1\|len=878 | 1.8990 | 3.7297 | 0.0000 | 0.0142 | - |
| TRINITY_DN10367_c0_g1_i1\|len=705 | 1.8851 | 3.6938 | 0.0004 | 0.0408 | - |
| TRINITY_DN24883_c0_g1_i1\|len=993 | 1.8803 | 3.6815 | 0.0005 | 0.0484 | - |
| TRINITY_DN1958_c0_g1_i1\|len=1067 | 1.8170 | 3.5235 | 0.0001 | 0.0222 | - |
| TRINITY_DN2195_c0_g1_i1\|len=984 | 1.8135 | 3.5149 | 0.0002 | 0.0323 | - |
| TRINITY_DN4459_c0_g1_i1\|len=671 | 1.7844 | 3.4447 | 0.0000 | 0.0116 | - |
| TRINITY_DN25548_c0_g1_i1\|len=605 | 1.7683 | 3.4066 | 0.0000 | 0.0016 | - |
| TRINITY_DN26460_c0_g1_i1\|len=1828 | 1.7633 | 3.3948 | 0.0000 | 0.0035 | - |
| TRINITY_DN18886_c0_g1_i1\|len=1301 | 1.7486 | 3.3603 | 0.0001 | 0.0149 | - |
| TRINITY_DN16759_c1_g1_i1\|len=778 | 1.7174 | 3.2885 | 0.0000 | 0.0015 | - |
| TRINITY_DN25703_c0_g1_i1\|len=836 | 1.7049 | 3.2600 | 0.0001 | 0.0157 | - |
| TRINITY_DN2450_c0_g1_i1\|len=1145 | 1.6939 | 3.2352 | 0.0001 | 0.0236 | - |
| TRINITY_DN24882_c0_g1_i1\|len=672 | 1.6823 | 3.2095 | 0.0002 | 0.0253 | - |
| TRINITY_DN28311_c0_g1_i1\|len=586 | 1.6814 | 3.2075 | 0.0002 | 0.0295 | - |
| TRINITY_DN28437_c0_g1_i1\|len=751 | 1.6777 | 3.1991 | 0.0000 | 0.0122 | - |
| TRINITY_DN2378_c0_g1_i1\|len=966 | 1.6483 | 3.1346 | 0.0001 | 0.0210 | - |
| TRINITY_DN3375_c0_g1_i1\|len=666 | 1.6317 | 3.0988 | 0.0000 | 0.0072 | - |
| TRINITY_DN26264_c0_g1_i1\|len=1966 | 1.6087 | 3.0497 | 0.0001 | 0.0233 | - |
| TRINITY_DN10179_c0_g1_i1\|len=561 | 1.6015 | 3.0345 | 0.0000 | 0.0123 | - |
| TRINITY_DN24095_c0_g1_i1\|len=782 | 1.5974 | 3.0259 | 0.0004 | 0.0415 | - |
| TRINITY_DN5007_c0_g1_i1\|len=878 | 1.5862 | 3.0026 | 0.0002 | 0.0310 | - |
| TRINITY_DN25025_c0_g1_i1\|len=836 | 1.5639 | 2.9564 | 0.0001 | 0.0149 | - |
| TRINITY_DN15907_c0_g1_i7\|len=747 | 1.5525 | 2.9333 | 0.0003 | 0.0366 | - |
| TRINITY_DN27976_c0_g1_i1\|len=1303 | 1.5521 | 2.9324 | 0.0001 | 0.0232 | - |
| TRINITY_DN3984_c0_g1_i1\|len=888 | 1.5338 | 2.8955 | 0.0001 | 0.0209 | - |
| TRINITY_DN16759_c0_g1_i1\|len=624 | 1.5251 | 2.8781 | 0.0000 | 0.0072 | - |
| TRINITY_DN210_c0_g1_i1\|len=688 | 1.5244 | 2.8766 | 0.0001 | 0.0176 | - |
| TRINITY_DN13753_c0_g1_i2\|len=786 | 1.5103 | 2.8486 | 0.0002 | 0.0265 | - |
| TRINITY_DN2474_c0_g1_i1\|len=565 | 1.4992 | 2.8269 | 0.0000 | 0.0026 | - |
| TRINITY_DN16759_c1_g2_i3\|len=773 | 1.4982 | 2.8249 | 0.0000 | 0.0102 | - |
| TRINITY_DN14163_c0_g1_i1\|len=520 | 1.4900 | 2.8088 | 0.0000 | 0.0015 | - |
| TRINITY_DN25705_c0_g1_i1\|len=1145 | 1.4613 | 2.7536 | 0.0001 | 0.0204 | - |
| TRINITY_DN5117_c0_g1_i1\|len=645 | 1.4600 | 2.7511 | 0.0000 | 0.0037 | - |
| TRINITY_DN25829_c0_g1_i1\|len=1134 | 1.4369 | 2.7073 | 0.0005 | 0.0490 | - |
| TRINITY_DN2954_c0_g1_i1\|len=2603 | 1.4270 | 2.6889 | 0.0005 | 0.0471 | - |
| TRINITY_DN19123_c0_g1_i1\|len=906 | 1.4215 | 2.6787 | 0.0000 | 0.0092 | - |
| TRINITY_DN20618_c0_g1_i1\|len=1399 | 1.4068 | 2.6514 | 0.0000 | 0.0002 | - |
| TRINITY_DN27809_c0_g1_i1\|len=651 | 1.4031 | 2.6446 | 0.0001 | 0.0144 | - |
| TRINITY_DN13417_c0_g1_i4\|len=1087 | 1.3942 | 2.6285 | 0.0000 | 0.0122 | - |
| TRINITY_DN16674_c0_g1_i5\|len=839 | 1.3940 | 2.6281 | 0.0002 | 0.0302 | - |
| TRINITY_DN21297_c0_g2_i1\|len=657 | 1.3838 | 2.6095 | 0.0000 | 0.0003 | - |
| TRINITY_DN27082_c0_g1_i1\|len=1478 | 1.3640 | 2.5740 | 0.0005 | 0.0487 | - |
| TRINITY_DN7210_c0_g1_i1\|len=608 | 1.2843 | 2.4357 | 0.0000 | 0.0008 | - |
| TRINITY_DN18230_c0_g1_i2\|len=693 | 1.2804 | 2.4290 | 0.0002 | 0.0248 | - |
| TRINITY_DN10171_c1_g1_i1\|len=1052 | 1.2720 | 2.4149 | 0.0000 | 0.0115 | - |
| TRINITY_DN4256_c0_g1_i1\|len=820 | 1.2597 | 2.3945 | 0.0000 | 0.0069 | - |
| TRINITY_DN9692_c0_g1_i1\|len=736 | 1.2496 | 2.3777 | 0.0001 | 0.0222 | - |
| TRINITY_DN14758_c0_g1_i2\|len=900 | 1.2456 | 2.3711 | 0.0000 | 0.0077 | - |
| TRINITY_DN19547_c0_g1_i1\|len=2183 | 1.2438 | 2.3682 | 0.0000 | 0.0023 | - |
| TRINITY_DN6951_c0_g1_i1\|len=724 | 1.2392 | 2.3607 | 0.0000 | 0.0069 | - |
| TRINITY_DN1605_c0_g1_i1\|len=663 | 1.2000 | 2.2974 | 0.0004 | 0.0445 | - |
| TRINITY_DN14758_c0_g1_i3\|len=803 | 1.1579 | 2.2314 | 0.0001 | 0.0176 | - |
| TRINITY_DN11130_c0_g1_i1\|len=741 | 1.1220 | 2.1764 | 0.0000 | 0.0062 | - |
| TRINITY_DN10276_c0_g1_i1\|len=636 | 1.1116 | 2.1608 | 0.0003 | 0.0330 | - |
| TRINITY_DN6826_c0_g1_i1\|len=667 | 1.0891 | 2.1274 | 0.0000 | 0.0035 | - |
| TRINITY_DN13861_c0_g1_i4\|len=1084 | 1.0818 | 2.1167 | 0.0000 | 0.0123 | - |
| TRINITY_DN3666_c0_g1_i1\|len=568 | 1.0812 | 2.1158 | 0.0000 | 0.0069 | - |
| TRINITY_DN18451_c0_g1_i1\|len=1615 | 1.0422 | 2.0593 | 0.0001 | 0.0217 | - |
| TRINITY_DN18364_c0_g1_i2\|len=1717 | 0.9770 | 1.9683 | 0.0003 | 0.0374 | - |
| TRINITY_DN13756_c0_g2_i2\|len=782 | 0.9767 | 1.9679 | 0.0001 | 0.0205 | - |
| TRINITY_DN14300_c0_g1_i1\|len=617 | 0.9678 | 1.9559 | 0.0002 | 0.0275 | - |
| TRINITY_DN8201_c0_g1_i1\|len=1048 | 0.9537 | 1.9368 | 0.0002 | 0.0303 | - |
| TRINITY_DN13840_c0_g1_i1\|len=805 | 0.9509 | 1.9331 | 0.0004 | 0.0459 | - |
| TRINITY_DN12552_c0_g1_i1\|len=1715 | 0.9252 | 1.8990 | 0.0001 | 0.0182 | - |
| TRINITY_DN19139_c1_g1_i1\|len=535 | 0.9168 | 1.8879 | 0.0003 | 0.0401 | - |
| TRINITY_DN18088_c0_g1_i6\|len=1389 | 0.9116 | 1.8812 | 0.0001 | 0.0149 | - |
| TRINITY_DN17362_c0_g1_i1\|len=810 | 0.8905 | 1.8538 | 0.0004 | 0.0425 | - |
| TRINITY_DN4034_c0_g1_i1\|len=1479 | 0.8712 | 1.8291 | 0.0005 | 0.0497 | - |
| TRINITY_DN11591_c0_g1_i1\|len=622 | 0.8288 | 1.7762 | 0.0002 | 0.0274 | - |
| TRINITY_DN12907_c0_g1_i2\|len=609 | 0.8230 | 1.7690 | 0.0002 | 0.0324 | - |
| TRINITY_DN12372_c0_g1_i1\|len=794 | 0.7954 | 1.7355 | 0.0004 | 0.0460 | - |
| TRINITY_DN2918_c0_g1_i1\|len=874 | 0.7920 | 1.7314 | 0.0004 | 0.0428 | - |
| TRINITY_DN19510_c0_g1_i1\|len=1395 | -0.7720 | -1.7076 | 0.0004 | 0.0455 | - |
| TRINITY_DN15353_c0_g1_i1\|len=1784 | -0.7913 | -1.7307 | 0.0002 | 0.0307 | - |
| TRINITY_DN22451_c0_g1_i1\|len=5590 | -0.9007 | -1.8669 | 0.0003 | 0.0393 | shaking B, isoform D |
| TRINITY_DN18709_c0_g1_i1\|len=1815 | -0.9155 | -1.8862 | 0.0002 | 0.0271 | pointed, isoform C |
| TRINITY_DN23462_c0_g1_i2\|len=3424 | -0.9656 | -1.9529 | 0.0004 | 0.0436 | - |
| TRINITY_DN19677_c0_g1_i1\|len=1975 | -0.9800 | -1.9725 | 0.0002 | 0.0277 | - |
| TRINITY_DN23873_c0_g1_i1\|len=6000 | -1.0328 | -2.0460 | 0.0001 | 0.0231 | - |
| TRINITY_DN22728_c0_g1_i6\|len=6297 | -1.0385 | -2.0540 | 0.0001 | 0.0230 | no receptor potential A, isoform E |
| TRINITY_DN22270_c0_g2_i1\|len=1857 | -1.0931 | -2.1333 | 0.0003 | 0.0334 | - |
| TRINITY_DN17875_c0_g1_i1\|len=1669 | -1.2050 | -2.3054 | 0.0001 | 0.0222 | - |
| TRINITY_DN20193_c0_g1_i1\|len=1784 | -1.2094 | -2.3124 | 0.0003 | 0.0405 | - |
| TRINITY_DN16130_c0_g1_i4\|len=2029 | -1.2111 | -2.3152 | 0.0003 | 0.0368 | - |
| TRINITY_DN18410_c0_g1_i1\|len=724 | -1.2451 | -2.3703 | 0.0001 | 0.0232 | - |
| TRINITY_DN20453_c1_g1_i2\|len=1480 | -1.3600 | -2.5669 | 0.0002 | 0.0253 | - |
| TRINITY_DN5413_c0_g1_i1\|len=922 | -1.3952 | -2.6302 | 0.0004 | 0.0408 | - |
| TRINITY_DN7898_c0_g1_i1\|len=1017 | -1.4301 | -2.6946 | 0.0001 | 0.0186 | protein kinase, cAMP-dependent, regulatory subunit type 2, isoform E |
| TRINITY_DN10287_c1_g1_i1\|len=613 | -1.4708 | -2.7718 | 0.0001 | 0.0186 | - |
| TRINITY_DN13361_c0_g1_i1\|len=916 | -1.5049 | -2.8380 | 0.0000 | 0.0036 | - |
| TRINITY_DN19695_c0_g1_i2\|len=2119 | -1.5078 | -2.8437 | 0.0001 | 0.0225 | - |
| TRINITY_DN19774_c0_g1_i1\|len=2408 | -1.5552 | -2.9388 | 0.0001 | 0.0217 | - |
| TRINITY_DN23630_c0_g1_i4\|len=921 | -1.5593 | -2.9472 | 0.0001 | 0.0231 | glutaminase, isoform F |
| TRINITY_DN14809_c0_g1_i2\|len=1009 | -1.5998 | -3.0311 | 0.0001 | 0.0209 | - |
| TRINITY_DN13185_c0_g1_i1\|len=815 | -1.6385 | -3.1134 | 0.0000 | 0.0055 | - |
| TRINITY_DN19411_c0_g1_i2\|len=1502 | -1.6481 | -3.1342 | 0.0001 | 0.0204 | - |
| TRINITY_DN16915_c0_g1_i2\|len=1362 | -1.6857 | -3.2171 | 0.0001 | 0.0225 | - |
| TRINITY_DN20944_c0_g1_i5\|len=1691 | -1.7361 | -3.3312 | 0.0003 | 0.0385 | - |
| TRINITY_DN23456_c1_g1_i6\|len=959 | -1.7369 | -3.3332 | 0.0001 | 0.0217 | - |
| TRINITY_DN13667_c0_g1_i1\|len=540 | -1.7754 | -3.4234 | 0.0004 | 0.0465 | - |
| TRINITY_DN3144_c0_g1_i1\|len=2344 | -1.7983 | -3.4782 | 0.0001 | 0.0222 | uncharacterized protein Dmel_CG34377, isoform C |
| TRINITY_DN12594_c0_g1_i2\|len=1019 | -1.8041 | -3.4922 | 0.0002 | 0.0260 | - |
| TRINITY_DN14673_c0_g1_i1\|len=2874 | -1.8680 | -3.6503 | 0.0001 | 0.0152 | - |
| TRINITY_DN13374_c0_g1_i2\|len=921 | -1.8729 | -3.6627 | 0.0000 | 0.0072 | - |
| TRINITY_DN10242_c0_g1_i1\|len=795 | -1.9415 | -3.8410 | 0.0004 | 0.0415 | - |
| TRINITY_DN25251_c0_g1_i1\|len=864 | -2.1913 | -4.5670 | 0.0003 | 0.0372 | - |
| TRINITY_DN24061_c0_g1_i1\|len=1097 | -2.2215 | -4.6639 | 0.0002 | 0.0309 | - |
| TRINITY_DN20529_c0_g1_i3\|len=1227 | -2.3324 | -5.0363 | 0.0001 | 0.0217 | - |
| TRINITY_DN3694_c0_g1_i1\|len=568 | -2.3396 | -5.0617 | 0.0004 | 0.0415 | - |
| TRINITY_DN13750_c0_g1_i1\|len=1005 | -2.3434 | -5.0749 | 0.0000 | 0.0033 | - |
| TRINITY_DN1798_c0_g1_i1\|len=626 | -2.4583 | -5.4958 | 0.0000 | 0.0115 | - |
| TRINITY_DN24579_c0_g1_i1\|len=698 | -2.5301 | -5.7760 | 0.0000 | 0.0072 | - |
| TRINITY_DN13384_c0_g1_i2\|len=1343 | -2.5698 | -5.9371 | 0.0000 | 0.0057 | pickpocket 17, isoform B |
| TRINITY_DN19378_c0_g1_i1\|len=1078 | -2.6648 | -6.3413 | 0.0003 | 0.0349 | uncharacterized protein Dmel_CG43085, isoform A |
| TRINITY_DN15945_c0_g1_i4\|len=1302 | -2.6861 | -6.4359 | 0.0000 | 0.0015 | nutrient amino acid transporter 1 |
| TRINITY_DN5919_c0_g1_i1\|len=562 | -2.9528 | -7.7425 | 0.0004 | 0.0419 | - |
| TRINITY_DN16219_c0_g1_i1\|len=1044 | -2.9876 | -7.9314 | 0.0001 | 0.0222 | - |
| TRINITY_DN27728_c0_g1_i1\|len=545 | -3.1496 | -8.8742 | 0.0000 | 0.0031 | - |
| TRINITY_DN1909_c0_g1_i1\|len=523 | -3.2812 | -9.7216 | 0.0000 | 0.0072 | - |
| TRINITY_DN3243_c0_g1_i1\|len=600 | -4.0901 | -17.0315 | 0.0000 | 0.0022 | - |
| TRINITY_DN4516_c0_g1_i2\|len=682 | -4.1402 | -17.6330 | 0.0000 | 0.0015 | - |
| TRINITY_DN4564_c0_g1_i2\|len=777 | -5.3755 | -41.5135 | 0.0000 | 0.0026 | - |
| TRINITY_DN16695_c0_g1_i2\|len=1845 | -6.2033 | -73.6844 | 0.0000 | 0.0002 | - |
